# Possible Mechanisms of String Formation in Complex Plasmas at Elevated Pressures

**DOI:** 10.3390/molecules26020308

**Published:** 2021-01-09

**Authors:** Victoria Yaroshenko, Mikhail Pustylnik

**Affiliations:** Institut für Materialphysik im Weltraum, Deutsches Zentrum für Luft- und Raumfahrt (DLR), 82234 Wessling, Germany; Mikhail.Pustylnik@dlr.de

**Keywords:** structural properties of fluids, plasma-related fluids, string fluids, complex plasmas, electrorheological plasmas, interaction potential

## Abstract

Possible mechanisms of particle attraction providing formation of the field aligned microparticle strings in complex plasmas at elevated gas pressures are theoretically investigated in the light of the Plasmakristall-4 (PK-4) experiment on board the International Space Station. The particle interaction energy is addressed by two different approaches: (i) using the dynamically screened wake potential for small Mach numbers derived by Kompaneets et al., in 2016, and (ii) introducing effect of polarization of the trapped ion cloud by discharge electric fields. Is is found that both approaches yield the particle interaction energy which is independent of the operational discharge mode. In the parameter space of the performed experiments, the first approach can provide onset of the particle attraction and string formation only at gas pressures higher than 40–45 Pa, whilst the mechanism based on the trapped ion effect yields attraction in the experimentally important pressure range 20–40 Pa and may reconcile theory and observations.

## 1. Introduction

For some years, numerous experiments have pointed out electrorheological properties of complex plasmas showing that small micrometer-sized grains introduced into the discharge plasma can form string structures aligned with external electric fields. Such a particle alignment has been observed in the sheath of radio-frequency (rf) discharges [1,2,3], striations of dc discharges and in the periphery of inductively coupled rf plasma [4]. Under microgravity conditions, the chain formations have been reported in a dilute plasma near the midplane of parallel-plate rf plasma in subsonic ion flows [5] and in the dc discharge of the Plasmakristall-4 (PK-4) facility [6].

The PK-4 instrument (see Ref. [7] for detailed description of the set up) operates under microgravity conditions, on board of the International Space Station (ISS) and its elongated working space inside the U-shaped glass tube is ideally suited for string observations in a wide range of plasma conditions. Another advantage of the PK-4 instrument is that additionally to the dc mode, it can be operated in an ac regime, e.g., the duty cycle of the polarity switching 50% produces a symmetric square-form variations of the discharge electric field $E_{0}\left(t\right)$ with the time-averaged field $\langle E_{0}\left(t\right)\rangle =0$. Changing the polarity between two electrodes typically occurs with the frequency in the range ∼300–500 Hz. Such frequencies are higher than typical particle plasma frequency, but smaller than the ion plasma frequency, so that only plasma ions react to the field modulation.

**Figure 1:**
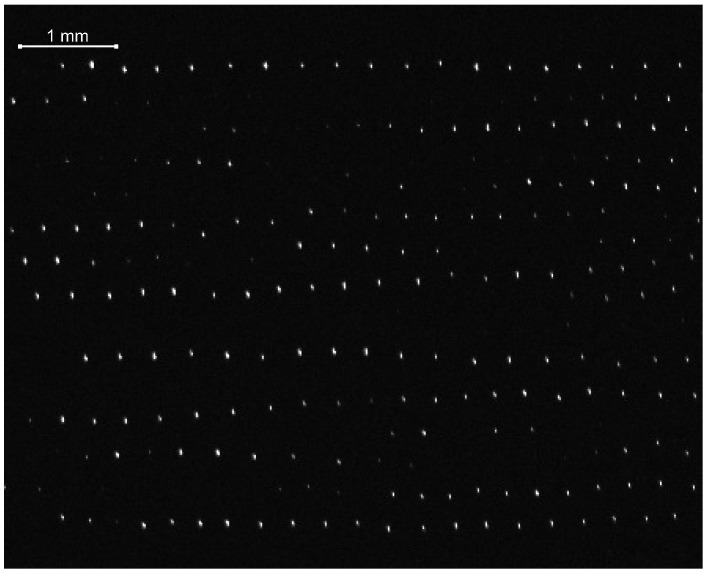
Image of the particle strings aligned with the discharge electric field in argon plasma of the Plasmakristall-4 (PK-4) chamber (electric current j=0.5 mA, polarity switching 50%, gas pressure 32 Pa, particle diameter 3.4 μm). The image is a vertical cross section through the particle cloud.

**Figure 2:**
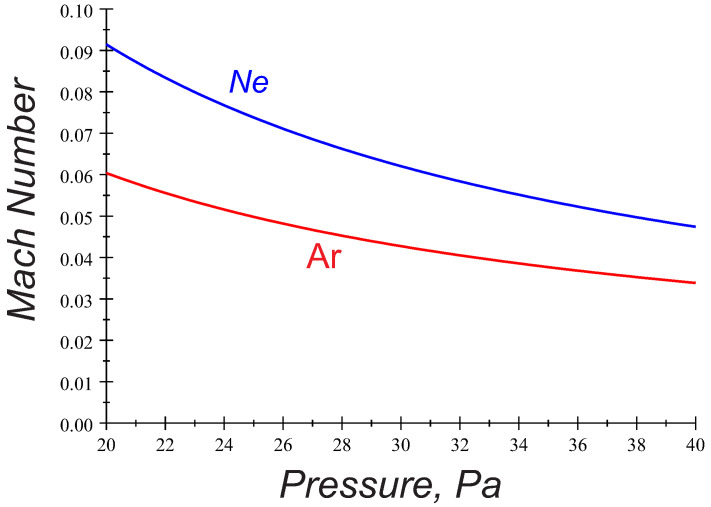
Mach number (ion drift velocity $u_{i}=eE_{0}/m_{i}\nu_{in}$ divided by the sound speed $\sqrt{T_{e}/m_{i}}$ ) versus gas pressure in argon (red) and neon (blue). Calculations have been done in the parameter space of the PK-4 facility, discharge current j=0.5 mA.

In the recent PK-4 experiments, the particle chains aligned with the axial discharge electric field are observed in the pressure range ∼20–40 Pa at both regimes of dc and ac fields mainly in Argon plasmas. In the ac mode, one often finds long particle strings consisting from more than a few tens of grains (Figure 1). The chain-like configurations seem to be stable with respect to perturbations in the longitudinal and transverse directions. The string formation occurs when the ion flow velocity is less (by a factor of 10 or more) than the sound speed. Shown in Figure 2 are the variations of the axial ion drift velocity $u_{i}$ (expressed as a Mach number $M=u_{i}/\sqrt{T_{e}/m_{i}}$) with gas pressure $p_{n}$. Here, we have assumed that $u_{i}≃eE_{0}/m_{i}\nu_{in}$, where the standard notations for the electron temperature $T_{e}$, ion mass $m_{i}$, and ion-neutral collision frequency $\nu_{in}$ are used. Calculations in Figure 2 and everywhere in the paper are done using the respective probe measurements of the discharge parameters made in the PK-4 chamber in the absence of dust [7,8,9,10].

In theoretical studies and simulations, the particle attraction providing the string configurations has been commonly attributed to the existence of a wake structure in the flowing plasma. The positive space-charge is accumulated due to the ion focusing in the immediate downstream region of a negatively charged grain. The downstream microparticle then experiences an attractive force towards the upstream particle wake, thus aligning grains along the ion flow direction. Many aspects of the ion wake formation and associated modifications of the particle interaction potential in various laboratory conditions have been studied theoretically, numerically and experimentally (see, e.g., References [11,12,13,14,15] and references herein). However, most of the theoretical results indicate that, to provide such an effective particle-wake interaction, the ion flow velocity should be in the order of the sound velocity. For example, a recent PIC simulation performed at low ion flow velocity showed that the neutral collisions suppress the wake charging mechanism, and the attractive part of the potential disappears when the ion flow decreases below a critical value, corresponding to the ion Mach numbers M∼0.4 [12]. The dust dynamics simulations with a dynamically screened Coulomb potential obtained from the linear response theory give the critical ion Mach numbers M∼0.1 [11]. As shown in Figure 2, these values, however, remain far above those observed in the PK-4 experiments, and numerous experiments, indicate that there exists a mechanism aligning grains even at slower ion flow velocities. Moreover, the application of the ac regime in the PK-4 set up eliminates the asymmetry effect associated with the downstream/upstream particle. The recent PK-4 observations, thus giving an extra impetus to explore possible mechanisms of string formation in the low-Mach-number domain and address two different discharge operational modes.

Considering particle interaction, it is usually very helpful to operate in terms of the particle potential, and we define it in two different ways. At first, we apply the analytical expression for the wake potential $\phi \left(r\right)$ around a negatively charged grain embedded in slowly flowing collisional plasmas [16]. The potential energy of a particle having charge *Q* in a potential created by another identical grain is given in a standard way $U=Q\phi \left(r\right)$. Another approach deals with the effect of trapped ions which become important in collisional plasmas and can dominate in the particle shielding [17]. Embedded even in weak electric fields, a grain “coated” by a trapped ion cloud is polarized, and its electrostatic potential $V\left(r\right)$ can be expanded in multipole moments [18]. As a result, the potential of a “coated” particle additionally includes contributions due to the field induced dipole and higher moments of the ion charge distribution within the shielding cloud. If the dipole moment strongly dominates, the particle potential energy can be approximated by

(1) $$W≃QV\left(r\right)-\sum_{\alpha }p_{\alpha }\frac{\partial }{\partial r_{\alpha }}V\left(r\right).$$

The first term corresponds to the energy of a particle charge *Q* in the potential $V\left(r\right)$, whilst the second one describes the electrostatic energy of the dipole in the electric field $\nabla V\left(r\right)$. Such an approach is reminiscent of the treatment of the intermolecular forces in classical electrodynamics [19].

Below, we discuss whether the two physically different mechanisms might provide the grain attraction and, thus, be responsible for the chain formation in the conditions relevant for the PK-4 experiments, considering both the dc and ac discharge modes.

## 2. Particle Potential in Collisional Complex Plasma with a Subsonic Ion Flow

We start with the theoretical expression for the grain potential $\phi \left(r\right)$ obtained in the frame of a self-consistent kinetic theory that incorporates the electric field, ion neutral collisions and associated modification of the ion velocity distribution [16]. Note that modification of this potential has been invoked in the stability analysis of particle pairs in weakly collisional plasmas [20]. Applying its general expression for the experimentally important case of large electron-to ion temperature ratio $\tau =T_{e}/T_{i}\gg 1$ and very low Mach number (M≪1), the long-range asymptotic ($\kappa =r/\lambda_{D}>1$) of the potential reads as

(2) $$\phi \left(\kappa \right)≃\frac{Q}{\lambda_{D}}\left(\frac{\exp\left(-\kappa \right)}{\kappa }-\frac{d}{\kappa^{2}}\cos\theta +d^{2}\frac{\left(2\omega_{pi}^{2}/\nu_{in}^{2}-1\right)}{\kappa^{3}}\left(3\cos^{2}\theta -1\right)+\ldots \right),$$

where $T_{i}$, $\lambda_{D}$, and $\omega_{pi}$ refer to the ion temperature, ion Debye length, and ion plasma frequency, respectively. The quantity θ denotes the angle between r and external field $E_{0}$, and *d* is defined through $d=M_{i}\nu_{in}/\omega_{pi}$ with the thermal ion Mach number given by $M_{i}=u_{i}/\sqrt{T_{i}/m_{i}}$.

The first term in (2) introduces a spherically symmetric screened Coulomb potential of the particle in the absence of ion flows. The second and third terms describe the dynamical wake structure induced by the external electric field $E_{0}$ and represent the dipole- and quadrupole-like contributions to $\phi \left(\kappa \right)$ reminding the standard expansion of an electrostatic potential in multipole moments. Note different dependencies of the last two terms on the gas pressure. The quadrupole contribution changes its sign at the gas pressure providing the condition $2\omega_{pi}^{2}/\nu_{in}^{2}=1$. In argon discharge of the PK-4 set up, this occurs at $p_{ncr}≃38\left(50\right)$ Pa (for the electric current 0.5 (1) mA, respectively). Hence, in the pressure range of a particular interest for the PK-4 experiments, $p_{n}\leq 40$ Pa, the quadrupole contribution in the longitudinal direction (θ=0) is mainly repulsive, while it becomes attractive in the transverse direction (θ=π/2). Furthermore, calculations for argon show that the dipole and quadrupole contribution in (2) are of the same order of magnitude at low gas pressure $p_{n}\ll p_{ncr}$, but already at $p_{n}>20$ Pa, the dipole term dominates providing a good convergence of the series (2).

**Figure 3:**
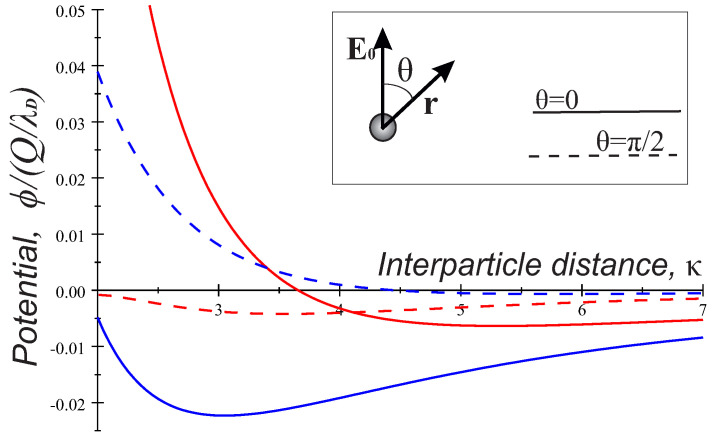
Longitudinal (solid) and transverse (dash) potential profiles $\phi \left(\kappa \right)/\left(Q/\lambda_{D}\right)$ in argon discharge, current j=0.5 mA, pressure $p_{n}=20$ Pa (red) and $p_{n}=30$ Pa (blue). Note a growth of the dipole contribution with pressure enhancement (solid curves).

In Figure 3, we give an example of the normalized potentials $\phi \left(\kappa \right)/\left(Q/\lambda_{D}\right)$ in the longitudinal (ion flow) direction (θ=0) and transverse direction (θ=π/2). Here, the calculations of the dipole and quadrupole terms were performed in the parameter space of the PK-4 set up in argon at the discharge current j=0.5 mA. It is seen that, in all cases, a repulsive region ($\phi \left(\kappa \right)>0$) at small κ≤ 2–4 switches to attraction ($\phi \left(\kappa \right)<0$) at longer distances. As has been mentioned, in the transverse case, the quadrupole term solely provides an attractive potential at pressures below $p_{ncr}∼38$ Pa. The transverse curve is typically shallower than the respective longitudinal one.

## 3. Discussion of the Particle Interaction in the Model of the Dynamically Screened Wake Potential

The energy of a grain carrying the charge *Q* in the electrostatic potential (2) is given by $U\left(r\right)=Q\phi \left(r\right)$, and *U* as a function of the interparticle distance has forms shown in Figure 3. As soon as the electric field is constant, in the longitudinal direction, $U\left(\kappa \right)$ displays an attractive region at the long range due to the charge-dipole interaction and a repulsive region at close range mainly due to the Coulomb interactions of like-charges. An attractive transverse interaction due to the charge-quadrupole interactions occur at pressures $p_{n}<p_{ncr}$, as has been discussed above. Such a behavior of $U\left(\kappa \right)$ might be relevant for the PK-4 observations in the dc regime.

We now consider the energy of interaction between two particles *i* and *j* that is $U=Q(\phi_{ij}+\phi_{ji})/2$, where $\phi_{ij}$ denotes the potential (2) created by the *i* particle at the position *j*. For two identical microparticles in the dc mode, the charge- dipole contributions ∝cosθ are canceled, and the pair interaction energy *U* is determined by the screened Coulomb and charge-quadrupole-like terms of Equation (2). It turns out that such *U* is described exactly as the time averaged interaction energy obtained below for the polarity switching regime. Indeed, in case of the ac mode, the fast (with respect to ion dynamics) polarity switching causes time variations of the induced dipole moment $p\left(t\right)\propto d\left(t\right)\propto E_{0}\left(t\right)$. At the duty cycle of 50%, the time-averaged values become $\langle E_{0}\left(t\right)\rangle =\langle d\rangle =0$ and $\langle d^{2}\rangle =d^{2}$, yielding the time averaged interaction energy

(3) $$\langle U\rangle ≃\frac{Q^{2}}{\lambda_{D}}\left[\frac{\exp\left(-\kappa \right)}{\kappa }+\frac{d^{2}}{\kappa^{3}}\left(2\omega_{pi}^{2}/\nu_{in}^{2}-1\right)\left(3\cos^{2}\theta -1\right)\right].$$

**Figure 4:**
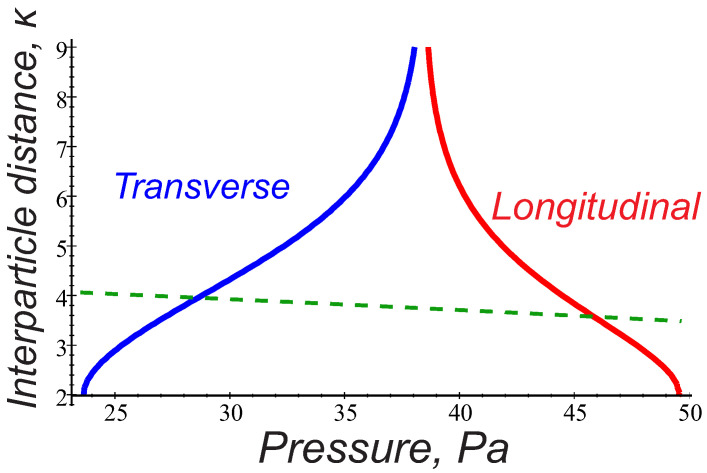
Solid curves express $\langle U\rangle =0$ in the parameter space $\left(p_{n},\kappa \right)$ for θ=0, (red) and θ=π/2, (blue). Particle attraction domain lies above the curves. Dashed curve expresses variations of $\kappa_{0}=r_{0}/\lambda_{D}$ for typical interparticle separation $r_{0}∼$ 300 μm observed in the PK-4 experiments. Calculations have been made for argon, j=0.5 mA.

Now, only the charge-quadrupole interactions can provide the mutual particle attraction. In Figure 4, we plot the attraction domain of the interaction energy (3) in the parameter space $\left\{p_{n},\kappa \right\}$ in argon gas at j=0.5 mA, considering two different grain orientations, θ=0 and θ=π/2, respectively. Quantities $p_{n}$ and κ lying above both curves lead to the grain attraction. The dashed curve expresses variations of $\kappa_{0}=r_{0}/\lambda_{D}$ corresponding to the typical interparticle distance $r_{0}∼300$ μm observed in the PK- 4 string experiments. As can be seen, the interaction energy (3) can hardly be responsible for the chain-like structures having the interparticle distances ∼300 μm and existing in the experimental range $p_{n}∼$ 20–40 Pa. Indeed, the averaged interaction energy $\langle U\rangle$ admits attraction at the pressure domain $p_{n}<30$ Pa in the transverse direction θ=π/2, but it cannot provide the grain alignment with the ion flow. On the other hand, the onset of the longitudinal attraction requires significantly higher pressure (in Figure 4 $p_{n}>45$ Pa). One has to conclude, therefore, that the considered mechanism fails to explain the chain-like configurations at the observed conditions, but might be important for the grain agglomeration either at low ($p_{n}<30$ Pa) or high ($p_{n}>$ 40–45 Pa) gas pressure.

## 4. Trapped Ions and Polarization of the Ion Cloud

Contrary to the dynamically shielded potential used in the previous section, another approach is based on the effect of trapped ions and polarization of the associated ion cloud by external electric fields. The standard charging models ignore plasma ions that are trapped in the Debye sphere surrounding a negatively charged grain. In collisional plasmas, however, a large negative charge carried by grains $Z_{d}=Q/e\gg 1$ can easily confine positive ions which lose their energy in collisions with neutrals [17,21]. There are two conditions ensuring large values of the trapped ion density [17]. First, the thermal energy of neutrals has to be small compared to the electron one, $T_{n}\ll T_{e}$ so that nearly all the new created ions are unable to escape from the potential well around the particle. Another requirement ${\left(a/\lambda_{D}\right)}^{2}\ll T_{n}/T_{e}$ provides that the newly born ions have significant angular momentum and do not fall immediately onto the grain. Both conditions are satisfied to good extent in the parameter space of the PK-4 experiments; hence, the effect of the trapped ion can be important.

Analytical modeling of the density and distribution function of the trapped ions by Lampe et al., in 2003, revealed the dominating role of the trapped ions in the particle shielding. It was found that, in the limit $l_{i}/\lambda_{D}\gg 1$ (with $l_{i}$ being the ion mean free path), the trapped ion density in the shielding cloud can locally be one order of magnitude higher than the untrapped ion density, and the trapped ion population can, thus, neutralize ∼40% of the particle charge. Growth of the plasma collisionality only slightly decreases this number due to the reduction of the grain floating potential, and hence the depth of the potential well, which traps the ions [17,18]. The recent PIC simulations in the discharge conditions close to the PK-4 experiments support this conclusion and yield the trapped ion population which neutralizes ∼30% of the particle charge in the shielding cloud at gas pressures ∼40–50 Pa [22].

A grain with a trapped ion cloud is similar to the classical atom in the sense that electrons bound in an atom cancel the charge of the nucleus within. Embedded even in weak external electric fields such a system reveals charge separation and appearance of nonzero dipole and higher moments describing a spatial distribution of the bound charges. Therefore, contrary to the first approach, where the particle interaction energy *U* is defined as a product of the dynamical potential and the particle charge, now, it additionally involves the dipole-dipole and higher moment interactions.

For complex plasmas, the main difficulties lie in estimating the multipole moments associated with the trapped ions. Recently, an iterative multipole expansion techniques has been applied for the particle potential in collisional complex plasmas that takes into account the trapped ion effect and slow ion flows [18,23]. It turns out that, in weak electric fields obeying $eE_{0}\lambda_{D}/T_{i}<1$, the long range multipole expansion of the potential converges fast and one can neglect the higher-order terms (see Figure 5 in Ref. [18]). In the PK-4 experimental parameter space, typically $eE_{0}\lambda_{D}/T_{i}<1$, and the discussion will be now limited to the largest dipole contribution describing the field induced polarization of the trapped ion cloud. In the first approximation, the particle potential is thus assumed to be

(4) $$V\left(\kappa \right)≃\frac{Q}{\lambda_{D}}\left(\frac{\exp\left(-\kappa \right)}{\kappa }-\frac{d_{tr}}{\kappa^{2}}\cos\theta \right).$$

For estimations of a dipole moment associated with the trapped ions $p_{tr}=Q\lambda_{D}d_{tr}$, we use the approximation $p_{tr}≃a\left[\mu m\right]\sqrt{l_{i}/\lambda_{D}}E_{0}\lambda_{D}^{3}$ that follows from expression (10) of Ref. [23], where we put A∼1 and $eE_{0}\lambda_{D}/b_{p}T_{i}\ll 1$. Note that such $p_{tr}$ is based on the self-consistent numerical model describing the polarization of a plasma around an isolated, highly charged particle in collisional limit and may be applicable in the case of low Mach numbers and τ≫1 (see Reference [23] for details).

**Figure 5:**
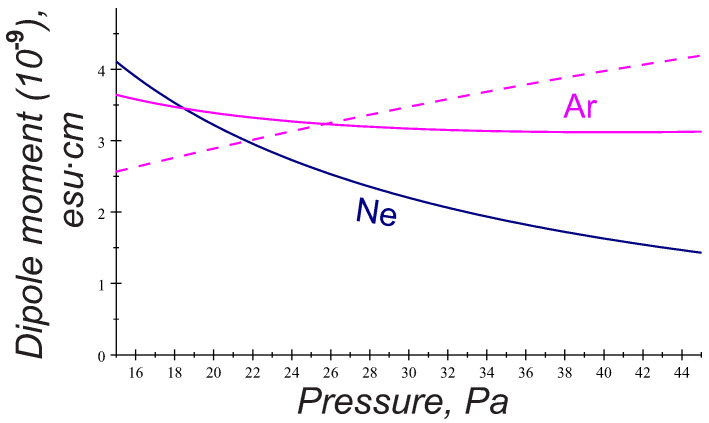
Dipole moment due to the trapped ions, acquired by particles (a=1.7 μm), versus gas pressure calculated for the PK-4 discharge in argon and neon, j=1 mA. For comparison the respective estimates of dipole moment $p=Q\lambda_{D}\left(eE_{0}\lambda_{D}/T_{i}\right)$ involved in (2) for argon discharge are given by dashed curve. Calculations employ particle charge estimations made for the PK-4 facility in Ref. [24].

It turns out that variations of the PK-4 discharge parameters with gas pressure provide the slow decreasing function $p_{tr}\left(p_{n}\right)$. This is illustrated in Figure 5, where the estimations of the dipole moment acquired by the particles of a=1.7 μm radius due to the trapped ions are shown as a function of gas pressure in argon and neon (solid curves). Note that the dipole moment $p_{tr}$ in neon yields a stronger reduction with gas pressure than in argon. For comparison, we also plot the dipole moment involved in the wake potential (2): $p=Q\lambda_{D}d$ (dashed curve). Interestingly enough, both models based on different physics give similar values of the induced dipole moment but display the inverse trends in the variations with gas pressure.

## 5. Discussion of the Particle Interaction Accounting for the Ion Trapping Effect

The energy of a grain “coated” by trapped ion cloud and immersed in a potential (4) is given by expression (1). This can be explicitly rewritten as

(5) $$W≃\frac{Q^{2}}{\lambda_{D}}\left[\frac{\exp\left(-\kappa \right)}{\kappa }-\frac{d_{tr}\cos\theta }{\kappa^{2}}\left(1-\left(1+\kappa \right)\exp\left(-\kappa \right)\right)-\frac{d_{tr}^{2}}{\kappa^{3}}\left(3\cos^{2}\theta -1\right)\right].$$

It should be stressed that, although mathematically the potential energy (5) and considered earlier potential (2), both admit a form similar to the multipole expansion, the terms involved in these expressions have different physical origins. Contrary to the first approach, where the free ions dynamically shield a grain and the wake charge distributions of the neighboring grains do not interact directly, the trapped ion clouds through the interactions of their dipole (and higher) moments can affect the potential energy (5).

Note the κ-dependence of various terms involved in (5). The charge-charge interaction is proportional to $\kappa^{-1}\exp\left(-\kappa \right)$, the charge-dipole to $\kappa^{-2}$, the dipole-charge to $\kappa^{-2}\left(1+\kappa \right)\exp\left(-\kappa \right)$, while the dipole-dipole to $\kappa^{-3}$. Due to the screening factor in the initial particle potential (2), the attractive charge-dipole $\propto \kappa^{-2}$, and dipole-dipole contributions $\propto \kappa^{-3}$ might become increasingly significant at long range (κ>1).

Similar to the first approach, calculating the pair interaction energy of two “coated” particles in the dc field rules out the charge-dipole terms, and the pair interaction energy is given by the same expression as the time averaged pair interaction energy in the polarity switching mode, *viz.*

(6) $$\langle W\rangle =\frac{Q^{2}}{\lambda_{D}}\left[\frac{\exp\left(-\kappa \right)}{\kappa }-\frac{d_{tr}^{2}}{\kappa^{3}}\left(3\cos^{2}\theta -1\right)\right].$$

**Figure 6:**
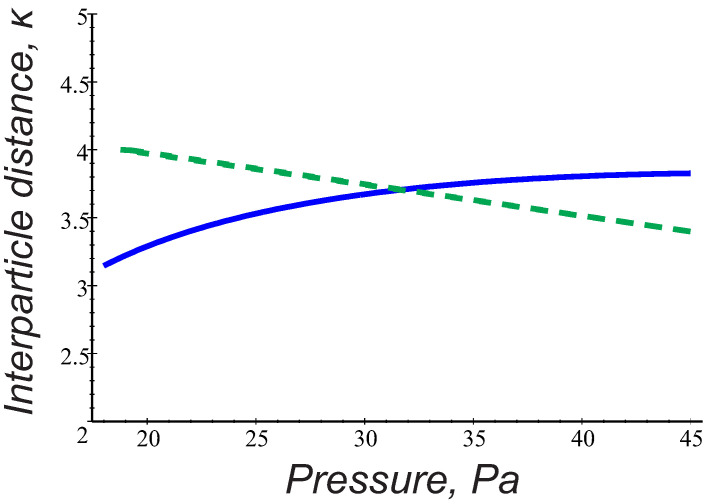
Longitudinal attraction domain in the parameter space ($p_{n},\kappa$) lies above the solid curve that expresses $\langle W\rangle =0$ for θ=0. Plasma parameters as in Figure 4, and the dashed curve displays variations of $\kappa_{0}=r_{0}/\lambda_{D}$ for typical grain separation $r_{0}∼300$ μm.

At this stage, it is interesting to further compare the interaction energy (6) to that one following from the wake potential (3) in the range of parameters relevant for the PK-4 experiments. It is clear that, in the case of trapped ions, the attraction mainly occurs when both particles are oriented along the ion flow direction (θ∼0), while (3) reveals possibility of the only transverse attraction, i.e., when the grains are radially displaced from the axis of the ion flow by the angles close to θ=π/2 (Figure 4). Moreover, the attractive terms in (6) result from the dipole-dipole interaction of the polarized trapped ion clouds, while, in (3), the attraction is of the charge-quadrupole interaction type. Keep in mind that the induced dipole moments in both mechanisms are of the same order $d∼d_{tr}$ (Figure 5), but the quadrupole term in (3) contains additionally a factor $\left(2\omega_{pi}^{2}/\nu_{in}^{2}-1\right)$ decreasing with pressure, so that the attractive mechanism due to the trapped ion effect appears to be more effective. This is also seen in Figure 6, where the solid curve expresses the condition $\langle W(p_{n})\rangle =0$ for θ=0, hence indicating the longitudinal attraction range in the parameter space $\left\{p_{n},\kappa \right\}$. Contrary to Figure 4, the admissible $p_{n}$ and κ lying above the solid curve arise in the direct vicinity of the observed particle separations $\kappa_{0}=r_{0}/\lambda_{D}$ (dashed curve) in the whole range of gas pressure 20–40 Pa.

**Figure 7:**
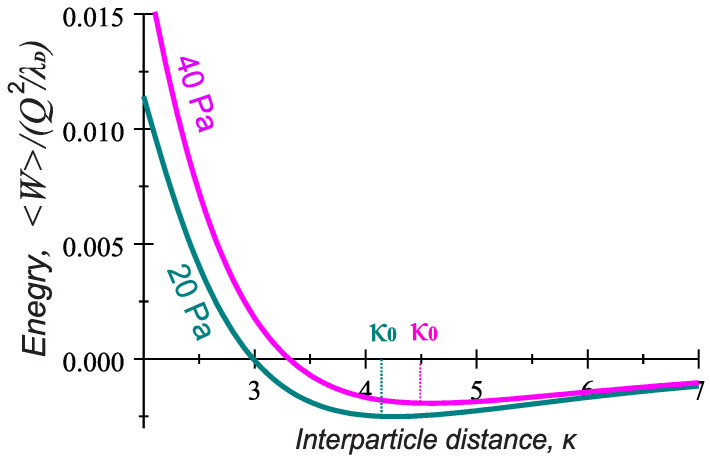
The time averaged interaction energy $\langle W\left(\kappa \right)/\left(Q^{2}/\lambda_{D}\right)\rangle$ versus the particle separation κ at different pressures 40 Pa (green-blue) and 20 Pa (magenta), j=1 mA. The quantity $\kappa_{0}$ indicates the equilibrium particle separation.

Finally, in Figure 7, we show the radial profiles of the longitudinal interaction energy (6). The examples where the gas pressure is chosen very close to lower and higher values from the experimental range of $p_{n}$ display a typical minimum of the interaction energy at $\kappa ∼\kappa_{0}∼4$. As the separation distance decreases below $\kappa_{0}$, the potential energy grows (indicating a repulsive force). However, at longer separation distances $>\kappa_{0}$, the energy is negative and approaches zero as the separation distance increases to $\kappa \gg \kappa_{0}$ (providing an attractive force). This indicates that at $\kappa ∼\kappa_{0}$, the particles experiences a zero force. If the two particles are further pressed together, past their equilibrium distance, repulsion begins to occur. At the point $\kappa ∼\kappa_{0}$, hence, the pair of particles is most stable and will remain in that orientation until an external force is exerted upon it. Calculations show that the obtained depth of the attractive potential well ${\langle W\left(\kappa_{0}\right)∼10^{-3}\left(Q^{2}/\lambda_{D}\right)\rangle }_{\max}$ is at least one order of magnitude larger than the thermal energy of the random motion of the particles at the room temperature $T_{d}≃0.03$ eV. Here, again, we have used the particle charges corresponding to a=1.7 μm estimated in the PK-4 set up in Ref. [24]. Larger grains have, therefore, even more chances to stay in the attractive potential well than smaller ones.

Figure 7 also shows that, in the considered range of gas pressure between 20 and 40 Pa, the longitudinal energy profiles lie close to each other, indicating that such changes in $p_{n}$ do not matter much. This is supported by estimates of the equilibrium interparticle distances $r_{0}∼\kappa_{0}\lambda_{D}∼250$ μm at $p_{n}=20$ Pa and $r_{0}∼280$ μm at $p_{n}=40$ Pa in the ac discharge mode.

## 6. Conclusions

To explore possible mechanisms of the microparticle attraction in collisional complex plasmas and hence shed a light on the string formation and transition to the string fluid state observed experimentally at very ion low Mach numbers, we theoretically analyzed the pair interaction energy addressing two approaches. The first one applies the theoretical model for the particle wake potential in collisional flowing plasmas [16], and another approach introduces the trapped ion distribution and associated dipole moment induced by external electric fields [18]. The results were then applied to the parameter space of the PK-4 facility on board ISS, using the probe measurements of the plasma parameters [7,8,9,10]. It was found that, in the experimental conditions, the local variations in the (free or trapped) ion charge distribution induced by weak discharge electric fields reduce significantly the repulsion between two particles, and it can be, furthermore, turned into explicit attraction at realistic interparticle distances. Interesting enough, both physically different models give similar values of the induced dipole moment that is only weakly varying with gas pressure. Moreover, both approaches yield the pair interaction energy which is independent of the operational discharge mode. The first approach provides onset of the field-aligning attraction due to charge–quadrupole interactions and may be important only at high gas pressures $p_{n}$ > 40–45 Pa, while the treatment based on the trapped ion effect provides the attractive part of the interaction energy along the ion flow in the whole experimental pressure range $p_{n}∼$ 20–40 Pa. Moreover, this approach can explain many experimentally observed features. Among them are:

1. The typical interparticle distances observed in the strings ∼250–300 μm correspond to the estimates of the equilibrium separation of two particles aligned with the ion flow (Figure 6).
2. Larger particle size is more favorable for the string formation.
3. The trend to form strings decreases with gas pressure and dc power (the dipole moment in Equations (5) and (6) is reduced with dc power and gas pressure).
4. Argon is more favorable for the string formation than neon (the dipole moment shows a stronger reduction with pressure in neon, than in argon; see Figure 5).
5. The particle longitudinal attraction in the dc and ac operational mode is found to be the same, but, in the PK-4 experiments, the string formation is observed more easily in the ac mode. One can assume that a short drift time of the particles in the dc field withing the camera field of view (∼1 s) might be too short compared to the time scales necessary for the string formation. In the ac mode, on the contrary, the microparticles remain almost stable in the camera field of view, and one can easily observe formation of the ordered field-aligned structures.

We conclude, therefore, that the approach invoked the trapped ion effect can reconcile qualitatively the theory and PK-4 observations. Since the string formation is quite generic in complex plasmas, the results presented here might also be applicable to other experiments performed in similar conditions. However, note that the used plasma parameters relate to the particle-free discharge plasmas and, thus, are not fully constrained by measurements yet. This means that a little stretching or new interpretation of particle attraction is possible.

## Sample Availability

Samples of the compounds are not available from the authors.
